# Gut microbiome-mediated epigenetic modifications in gastric cancer: a comprehensive multiomics analysis

**DOI:** 10.3389/fcimb.2025.1585881

**Published:** 2025-10-16

**Authors:** Zixing Qian, Wei Bai, Jiaxuan Li, Xianjun Rao, Guodong Huang, Xueping Zhang, Wenyu Wu, Jiabao Liu, Wei Wei

**Affiliations:** ^1^ College of Traditional Chinese Medicine, Hubei University of Chinese Medicine, Wuhan, Hubei, China; ^2^ Wangjing Hospital, China Academy of Chinese Medical Sciences, Beijing, China

**Keywords:** epigenetics, gut microbiome, gastric cancer, hub genes, miRNAs, transcriptomics sequencing

## Abstract

**Background:**

Gastric cancer (GC), a malignant and highly proliferative disease, has profoundly impacts a substantial global population and is associated with several variables, including genetic, epigenetic, and environmental impacts. Global variance is associated with *Helicobacter pylori* infection and dietary factors.

**Objectives:**

The aim of the present study was to understand and identify key genes significantly modulated by epigenetic changes that can serve as biomarkers and therapeutic targets for gastric cancer.

**Methods:**

This study employed an integrative multiomics approach to investigate gut microbiome-mediated epigenetic modifications in gastric cancer by utilizing publicly available transcriptomic and DNA methylation datasets, Quality control, normalization and deferentially expressed gene analysis of sequencing data were performed via standard bioinformatics pipelines. Functional enrichment analyses, including GO and KEGG pathway mapping, were performed to elucidate the biological pathways influenced by these interactions and network analysis was conducted using Cytoscape to identify hub genes. We conducted *in vitro* assays using the gastric adenocarcinoma cell lines AGS and MKN45, and the normal gastric epithelial cell line GES-1. The expression of selected candidate genes was evaluated using real-time PCR in these cell lines.

**Results:**

The GEO2R and coexpression network analyses revealed that six genes MAPK1, NOXO1, CUL1, CDK1, CDK2, and CCNB1 were significantly altered by modified DNA methylation and mRNA expression in GC. Owing to their identification across all epigenetic, transcriptomic, and miRNA datasets, we have designated these genes as shared genes. The results showed that the relative gene expression levels of MKN45 and AGS cell lines were higher than those in the GES-1 cell line in the control., and the results were aligned with the *in silico* findings.

**Conclusions:**

CDK1, CDK2, NOXO1, CUL1, MAPK1, and CCNB1 play pivotal roles in GC carcinogenesis and hold promise as early diagnostic biomarkers and therapeutic targets for GC.

## 1 Introduction

Gastric cancer (GC) is characterized as the principal epithelial malignancy originating from the stomach, and it represents a complex and heterogeneous illness with various risk factors (Smyth et al., 2020; Thrift and El-Serag, 2020). Despite its general decline in incidence and mortality across several countries in recent decades, GC is the fifth most prevalent malignancy and the fourth foremost cause of cancer-related mortality worldwide (Daniyal et al., 2015; Rawla and Barsouk, 2019). Despite a notable decline in the global burden of gastric cancer, it continues to be severe in specific regions, particularly Asia (Lordick et al., 2017). In recent decades, the association between the gut microbiota and cancers has been progressively elucidated, prompting investigations into the molecular mechanisms of the microbiome in cancer and its practical applications. Additionally, Manzoor et al. delineated three tiers of interaction between the microbiome and cancer: primary, secondary, and tertiary relationships, categorized by the proximity of tumors to pertinent bacteria (Elinav et al., 2019; Manzoor et al., 2020). While the majority of research has focused on colorectal cancer (CRC), an increasing number of studies in the past decade have indicated that intestinal microbes influence the progression of gastric cancer (GC) by modulating metabolism and immune signaling (Wang et al., 2023).

Recent studies have progressively emphasized the potential relevance of bacteria, other than *Helicobacter pylori*, in gastric cancer due to advancements in metagenomics, suggesting the possible application of the gut microbiota in this context. The composition of the gut microbiota in patients with GC can be affected by factors such as origin, pathogenic type, phase, and treatment (Liou et al., 2020). *H. pylori* infection is regarded as the primary risk factor for GC, but additional risk factors include Epstein–Barr virus (EBV) infection, a high-salt diet, tobacco use, and genetic predisposition, which lead to complicated interactions (Fakharian et al., 2022). In addition to these documented bacterial sensing pathways, microbial signals influence host physiology via epigenetic alterations that adjust gene expression without changing the genetic code. These microbiota-sensitive epigenetic changes include DNA and histone modifications, and their regulation by noncovalent epigenetic mechanisms such as long-noncoding RNAs and microRNAs(miRNA), also plays a role in initiating and sustaining epigenetic modifications (Matson et al., 2021; So et al., 2021). Epigenetic regulation is a powerful way by which the microbiota impacts the physiology of the host, influencing chemical donors for DNA or histone changes, modulating enzyme expression, or activating fundamental host-cell activities (Pepke et al., 2024). Additionally, comparative metagenomic analyses in humans, along with the identification of species-specific epigenetic alterations, indicate that the proliferation of various microbial species can influence unique gene expression profiles (Angers et al., 2020).

Although significant attention has given to short-chain fatty acids (SCFAs), multiomics methodologies have revealed that the microbiota generates a variety of bioactive metabolites that can affect epigenetic alterations and the influence on the host epigenome (Den Besten et al., 2013). The connections between microbiota composition and epigenetic alterations in inflammatory bowel disease (IBD), cancer, and obesity underscore the potential of epigenetic patterns as diagnostic instruments, linking genetic susceptibility and microbial dysbiosis to disease pathogenesis (Woo and Alenghat, 2022). Ultimately, the genetic modification of epigenetic-modifying bacteria or the management of microbe-derived epigenetic substrates may provide tailored prebiotic or probiotic therapy, providing localized control over epigenetic enzyme activity in the intestine (Gago et al., 2008; Reva et al., 2023).

This study aims to investigate the impact of gut microbiome-mediated epigenetic modifications in GC via a comprehensive multiomics approach that focuses on characterizing microbiome composition, identifying epigenetic changes, integrating multiomics data, assessing correlations with clinical outcomes, and exploring therapeutic prospects.

## 2 Materials and methods

**Figure 1:**
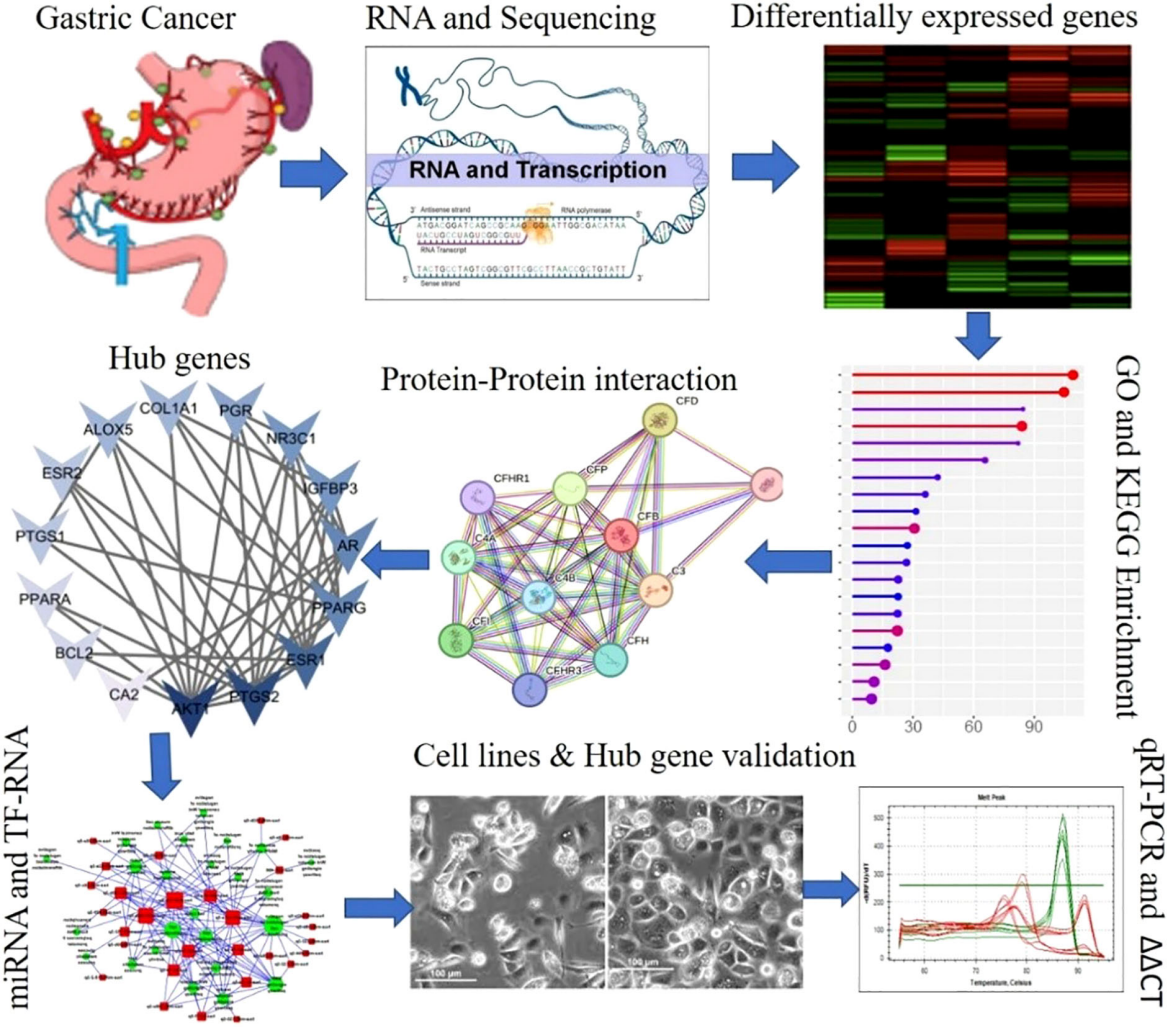
Graphical representation of the overall experimental design.

A graphical representation of the overall experimental design is shown in **Figure 1**.

### 2.1 Composition of the gut microbiome in GC patients

Newer stomach microbial analysis techniques have improved the understanding of the gastric microbiota and its composition. However, most studies have focused on intestinal-type adenocarcinoma, possibly because of the association of *H. pylori* with intestinal-type gastric cancer. We have mined the data and assembled the composition of the microbiota associated with GC via various tools such as PubTator, LitSuggest, and TeamTat. The key terms used for mining the data were microbiome, gastric cancer, epigenetics, and oncology, and finally datasets reported in the certain studies were selected specifically associated with GC, epigenetics and the microbiome. PubTator is a text-mining program designed for annotating all PubMed articles with essential biological elements (Wei et al., 2024). LitSuggest is an online platform for literature triage and document classification that uses artificial intelligence and machine learning (Allot et al., 2019, Allot et al., 2021). TeamTat is an online text annotation tool for biological texts and other domains (Islamaj et al., 2020).

### 2.2 Acquisition of transcriptomic datasets associated with GCs and the microbiome

The datasets associated with GC, epigenetics and the microbiome were retrieved from the Gene Expression Omnibus (GEO) and GEO2R was used to perform the gene expression analysis of the microarray/RNA-seq datasets. GEO2R is an online tool provided by NCBI GEO that allows users to compare gene expression across different conditions in a dataset without requiring coding expertise. The raw gene expression data from the GEO dataset (GSE30601 for methylation profiling, GSE39600 for *H. pylori*-mediated epigenetic dysregulation, and GSE58004 for the epigenetic associations during chronic *H. pylori* infection) were downloaded in series matrix format via the GEOquery package (v3.2) in R. Identifiers for genes were mapped to official gene symbols bases of the platform annotation file, and identifiers matching multiple genes were removed, while the average expression value was calculated for genes represented by multiple probes. Genes with low expression across all samples (expression values below the 20th percentile) were filtered out to reduce noise. Data normalization and differential gene expression analysis were conducted via the limma empirical Bayes method, with *p*-values adjusted for multiple testing via the Benjamini–Hochberg false discovery rate (FDR) method. Genes with an adjusted *p*-value < 0.05 and an absolute log2 fold change (|log2FC|) ≥ 2 were considered statistically significant and biologically relevant (Clough et al., 2023). GEO2R employs GEOquery and limma to conduct differential expression analysis via processed data tables provided by the original submitter as input. Consistent DEGs were identified across the three datasets.

### 2.3 Functional and pathway enrichment analysis

The Database for Annotation, Visualization, and Integrated Discovery (DAVID, version 6.8) was used to perform Kyoto Encyclopedia of Genes and Genomes (KEGG) pathway analysis as well as Gene Ontology (GO) analysis of the differentially expressed genes (DEGs). A *p-*value of <0.05 was set as the significance threshold. The DAVID is a widely used bioinformatics tool for functional enrichment analysis of gene lists and helps to identify the biological meaning behind DEGs by providing GO and KEGG pathway analyses (Huang et al., 2008; Huang et al, 2009).

### 2.4 Protein-protein interaction network and module analysis

A protein-protein interaction (PPI) network of the common genes in the four datasets was constructed and examined via the STRING database which is an online widely used resource for exploring PPIs (Szklarczyk et al., 2021). The default parameter values used were network edges, text mining, experiments, databases, coexpression, neighborhood, gene fusion, and co-occurrence as active interaction sources, and medium confidence (0.4) as the minimal necessary interaction score were among the default parameter values used. Cytoscape software was then used to visualize the PPI network (Shannon et al., 2003). In a PPI network, a node’s degree is defined as how many interactions it has with other nodes. In the PPI network, key nodes (genes/proteins) were chosen on the bases of degree > 15. With the use of the MCODE plugin and cluster modules, the network’s primary components were built (Bader and Hogue, 2003) following the import of the TSV data files to Cytoscape. The KEGG signaling pathway was initially utilized to annotate the core network modules, followed by in-depth analysis via the R programming language to identify the genes associated with these modules. Differences for which the *p* value was <0.05 were considered to be statistically significant (Kwak, 2023).

### 2.5 Screening of hub genes and analysis

Hub genes tend to have high connectivity within the network, meaning that they interact with multiple other genes and may be involved in key regulatory or signaling pathways (Bano et al., 2022). The cytoHubba plugin under Cytoscape is a prevailing means for categorizing important nodes and subnetworks in complex biological networks. It offers a user-friendly interface for examining important components in networks that depict gene controls, biological pathways, signal transductions, and protein–protein interactions. It carries involves a range of topological analysis algorithms, such as degree, Euclidean maximum neighborhood component (MNC), percolated component (EPC), maximum neighborhood component density (DMNC), maximum click centricity (MCC) and six centricity measures, including bottleneck, eccentricity, entrainment, centricity, intermediacy, and stress (Chin et al., 2014). These algorithms play important roles in elucidating the centrality of genes within biological networks. Researchers have identified genes that are important for network integrity and function.

### 2.6 Prediction and analysis of transcription factor and miRNA networks

Transcription factors and miRNAs form a complex regulatory network that finely controls gene expression in various biological processes and responses (Martinez and Walhout, 2009). During mammalian development and homeostasis in adult tissues TFs are invariably the primary factors involved in cell fate decision-making processes and lead to disease (Lee et al., 2024). The center was built by the TF gene interaction network analyzer for the gene. Network analyst is a web domain that provides comprehensive networks for gene expression visualization. TFs were obtained from the gene–gene network. JASPAR consists of a networking platform that analyses networks (Castro-Mondragon et al., 2022). TFs are proteins that can actively promote DNA replication. They are either sure that DNA replication is either started or suppressed before starting. In other words, they ensure that the cell will begin or not begin the process of making new copies of its DNA (Shiroma et al., 2020). On the other hand, miRNAs regulate gene expression after transcription has already started. They can regulate protein synthesis, usually by binding to miRNAs, preventing their translation into proteins (O’brien et al., 2018). The network of TF-miRNA regulators, which represent the hub genes, was constructed via RegNetwork (Liu et al., 2015). For the hub genes, the TF–miRNA regulatory network was constructed through the Network Analyst platform via the Reg Network repository. The network was cut off at 1°. Finally, the network was downloaded from Network Analyst and visualized via Cytoscape software.

### 2.7 Validation of the hub genes

Cell culture, total RNA extraction and real-time PCR analysis were carried out as described by Seo et al (Seo et al., 2023). To experimentally validate the bioinformatically identified hub genes, we conducted *in vitro* assays using the gastric adenocarcinoma cell lines AGS and MKN45, and the normal gastric epithelial cell line GES-1. *H. pylori* (strain NCTC11639, BNCC339501; BeNa Culture Collection) was cultured microaerobically (10% CO2, 5% O2, 85% N2) on Columbia blood agar plates for 3–4 days, followed by expansion in brain heart infusion broth to the stationary phase. Bacteria were harvested and resuspended in saline to a concentration of 1×10^8^ CFU/mL. The cells were maintained in RPMI-1640 medium supplemented with 10% fetal bovine serum and 1% penicillin–streptomycin. The cells were divided into three treatment groups: (1) control (normal culture medium); (2) *H. pylori* coculture (MOI = 100, 24 hours);and (3) 5- aza-2’-deoxycytidine (5-Aza-dC; MedChemExpress, Cat. No. HY-10586) pretreatment (10 μM, 24 hours) followed by *H. pylor* coculture. Total RNA was extracted via TRIzol reagent (Invitrogen, Cat. No. 15596026) according to the manufacturer’s instructions. The RNA concentration and purity were determined via a NanoDrop 2000 (Thermo Scientific). cDNA was synthesized via a RevertAid First Strand cDNA Synthesis Kit (Thermo Scientific, Cat. No. K1622). qPCR was performed via SYBR Green qPCR Mix (Biosharp, Cat. No. BL698A) on a StepOne Plus Real-Time PCR System (**Table 1**). The primer sequences were synthesized by Genewiz (China). Three duplicate wells were performed for each group, and each test was repeated three times. The average of the results was taken. Gene expression was normalized to that of GAPDH and was analyzed via the 2–ΔΔCt method.

**Table 1:** List of primers used in this study.

Name of gene	Sequence of primer	Product size
MAPK-1	F. TAGGTCTGGTGCTCAAAGGG R. CGCTACACCAACCTCTCGTA	121
NOXO-1	F. GCATCGAGAAGCTTTGGGAG R. GAGTTGGGACGAATTCAGGC	101
CUL1	F. CACAGTATCGAGCCAGCAAC R. GCTTTGTGGCTGCTCTTGAT	135
CDK 1	F. TCAGTGCCATTTTGCCAGAA R. AGCCTAGCATCCCATGTCAA	125
CCNB 1	F. CATGGTCTCCTGCAACAACC R. TGAGGAAGAGCAAGCAGTCA	140

## 3 Results and discussion

### 3.1 Gut microbiome composition in GC patients

The human microbiota contributes to sustaining physiological conditions and is implicated in diseases such as diabetes, obesity, allergies, atopic disorders, and cancer (Wang et al., 2023). The composition of host microorganisms, their functions, and their impact on illness progression, epigenetics and treatment are key elements to investigate. Various tools, such as PubTator, LitSuggest, and TeamTat, have revealed that the majority of the gut microbiota associated with GCs and epigenetics consists of *Helicobacter* spp., *Streptococcus anginosus*, *Proteobacteria* spp., *Prevotella melaninogenica, Propionibacterium acnes*, *Neisseria* spp.*, Enterococcus* spp., *Lactobacillus* spp.*, Firmicutes(*synonym *Bacillota*) spp.*, Bacillus* spp.*, Parasutterella* spp.*, Fusobacterium* spp.*, Brevibacillus* spp., *Enterobacter* spp.*, Cloacibacterium* spp. and *Suterella* spp. (**Supplementary Table S1**). The literature reveals that the gut microbiome is related mostly to *H. pylori* because of its crucial role in the development of GC. Nonetheless, the enrichment and diversity of other bacteria that can influence the tumor microenvironment are implicated in the course of GC and the effectiveness of immunotherapy.

### 3.2 Identification of DEGs

**Figure 2:**
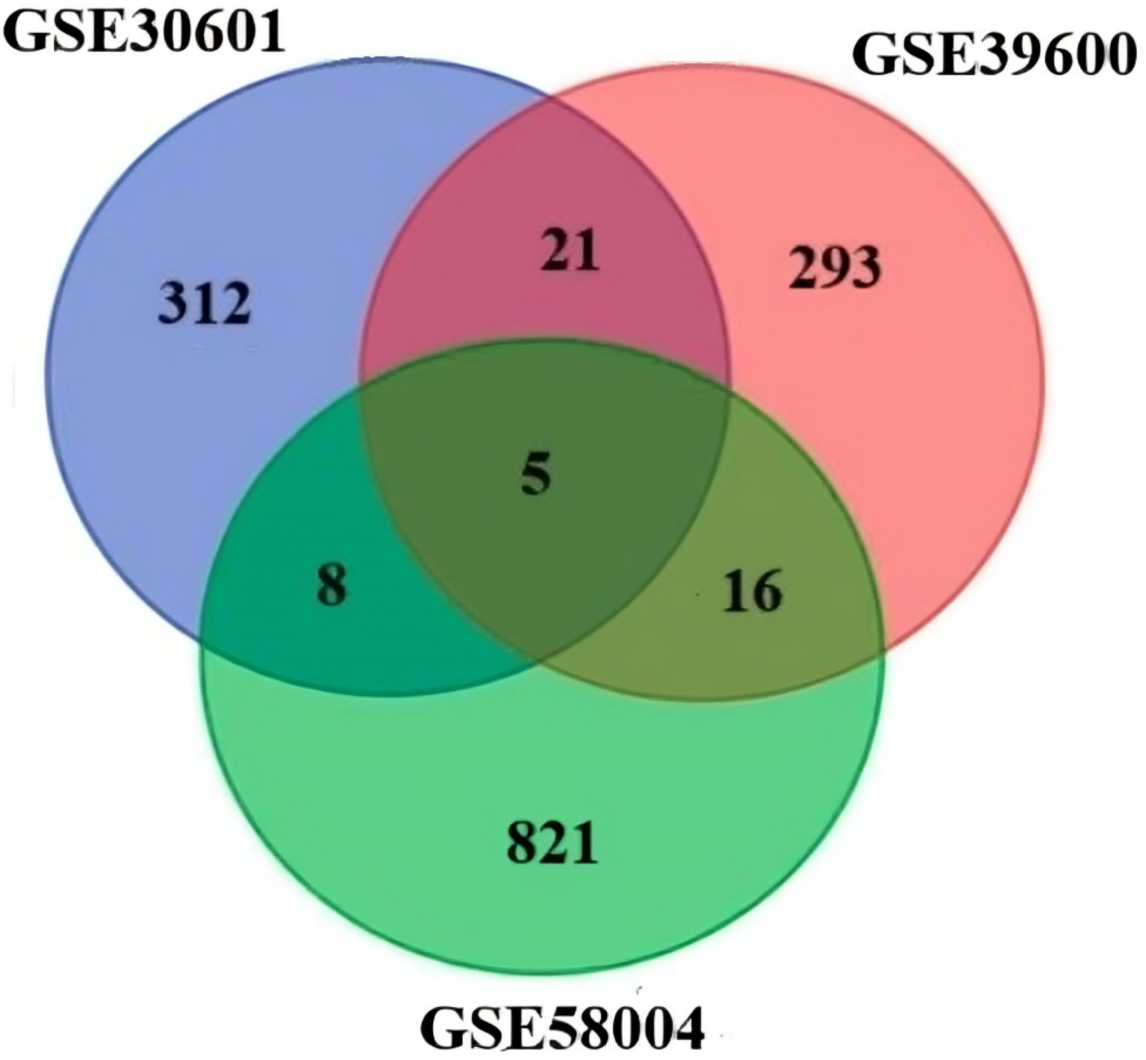
Venn diagram of the DEGs in the three datasets (DNA methylation datasets GSE58004, and GSE3900, representing epigenetic dysregulation by *H. pylori* and mRNA GSE58004 representing miRNA GC methylation).

On the basis of the composition, the following datasets were selected: the dataset GSE30601 which represents, methylation profiling via an array with 94 matched nonmalignant gastric and 204 gastric tumor samples; the GSE39600 dataset, which represents *H. pylori* mediated FOXD3 tumor-suppressive cascade epigenetic dysregulation; and the GSE58004 dataset, which represents epigenetic associations during chronic *H. pylori* infection. Significantly, DEGs were selected according to the following criteria: *p*value ≤ 0.05 and [log2FC] ≥ 0.5 (**Figure 2**). The DEGs identified were as follows: 312 differentially methylated genes in GSE30601, which represent the methylation profiling of GC; 293 in GSE39600; and 431 in GSE58004. The aggregate counts of downregulated and upregulated genes in each dataset are presented in **Table 2**. A comprehensive analysis of the three datasets revealed that six shared genes (CDK1, CDK2, NOXO1, CUL1, MAPK1, and CCNB1) were linked to GC (**Figure 2**).

**Table 2:** Summary of the microarray datasets and differentially expressed genes associated with the microbiota and gastric cancer in each dataset.

Sr. no.	Accession no.	Datasets	Upregulated genes	Downregulated genes	Total DEGs
1	GSE30601	Methylation Profiling of GC	217	129	346
2	GSE39600	Epigenetic Dysregulation by Hp	251	84	335
3	GSE58004	miRNA GC Methylation	363	97	460

Integrative bioinformatics analyses are evolving as the most obvious technique to illuminate the activities of the tumor microenvironment and uncover the critical genes and signaling pathways underlying disease pathogenesis. This method is extensively employed to identify significant biomarkers and treatment targets for different malignancies. Our study integrates the expression profiles of three key datasets of gastric cancer, microbiota and epigenetic modifications across healthy and GC samples by utilizing individual microarray datasets from NCBI-GEO (Zhang et al., 2019; Jin and Qin, 2020). The objective is to thoroughly elucidate the epigenetic modifications of GC and to identify pivotal hubs and common genes that significantly contribute to the development and progression of GC tumors. Overall, these results reinforce the complexity of the gut microbiome in GC beyond *H. pylori*, suggesting that a broader range of microbial species could be involved in modulating the tumor microenvironment.

### 3.3 Gene ontology and pathway enrichment analyses

**Figure 3:**
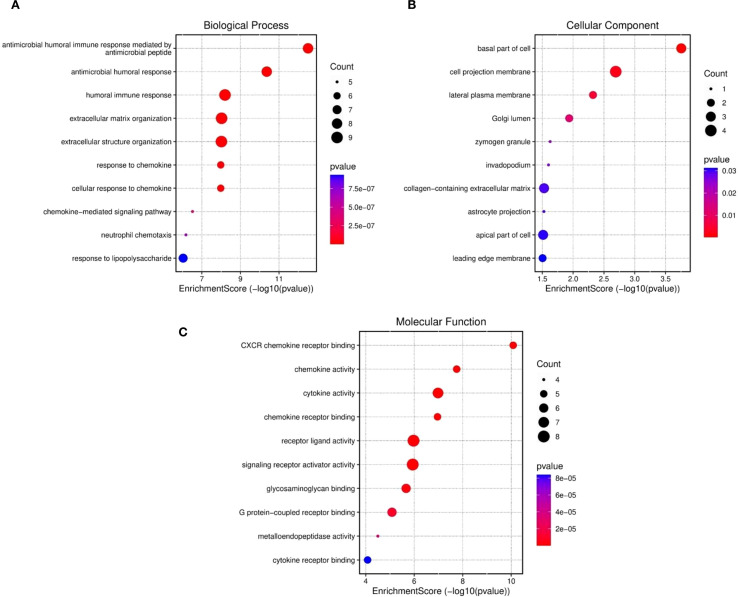
Functional enriched terms for biological processes, cellular components, and molecular functions across the three datasets were retrieved: **(A)** GO biological process, **(B)** cellular components and **(C)** molecular functions and.

The results retrieved from the Gene Ontology (GO) analysis revealed comprehensive functional enrichment. For example, upregulated genes associated with biological processes were mostly associated with the cell cycle, p53 pathway, apoptosis, and inflammatory pathways, which are prevalent (**Figure 3A**). The antimicrobial humoral response, extracellular structure organization, humoral immune response, extracellular matrix organization, chemokine response, and cellular response to chemokines. The gene set is highly enriched in immune-related pathways, suggesting that genes play roles in host defense, antibody-mediated responses, and chemokine signaling (Cui et al., 2014).The cellular components included the cell projection membrane, mainly plasma membrane, Golgi lumen, and zymogen granules (**Figure 3B**). The gene products function as signaling molecules (chemokines/cytokines) or receptors that mediate immune communication (Su et al., 2024). The majority of those enriched in molecular functions, however, were enriched in cytokine activity, chemokine receptor binding, receptor-ligand activity, CXCR, signaling activator, and glygamenchose activity (**Figure 3C**). The presence of apoptosis and p53 pathway involvement may indicate a cellular response to stress, potentially linking these findings to cancer or immune-related conditions (Pflaum et al., 2014). The extracellular matrix and structural organization findings suggest possible tissue remodeling, which could be relevant in fibrosis, tumor metastasis, or immune cell infiltration (Yuan et al., 2023). The functional enrichment suggested a strong immune–inflammatory response, which could be relevant to disease progression, the infection response, or cancer immunology.

**Figure 4:**
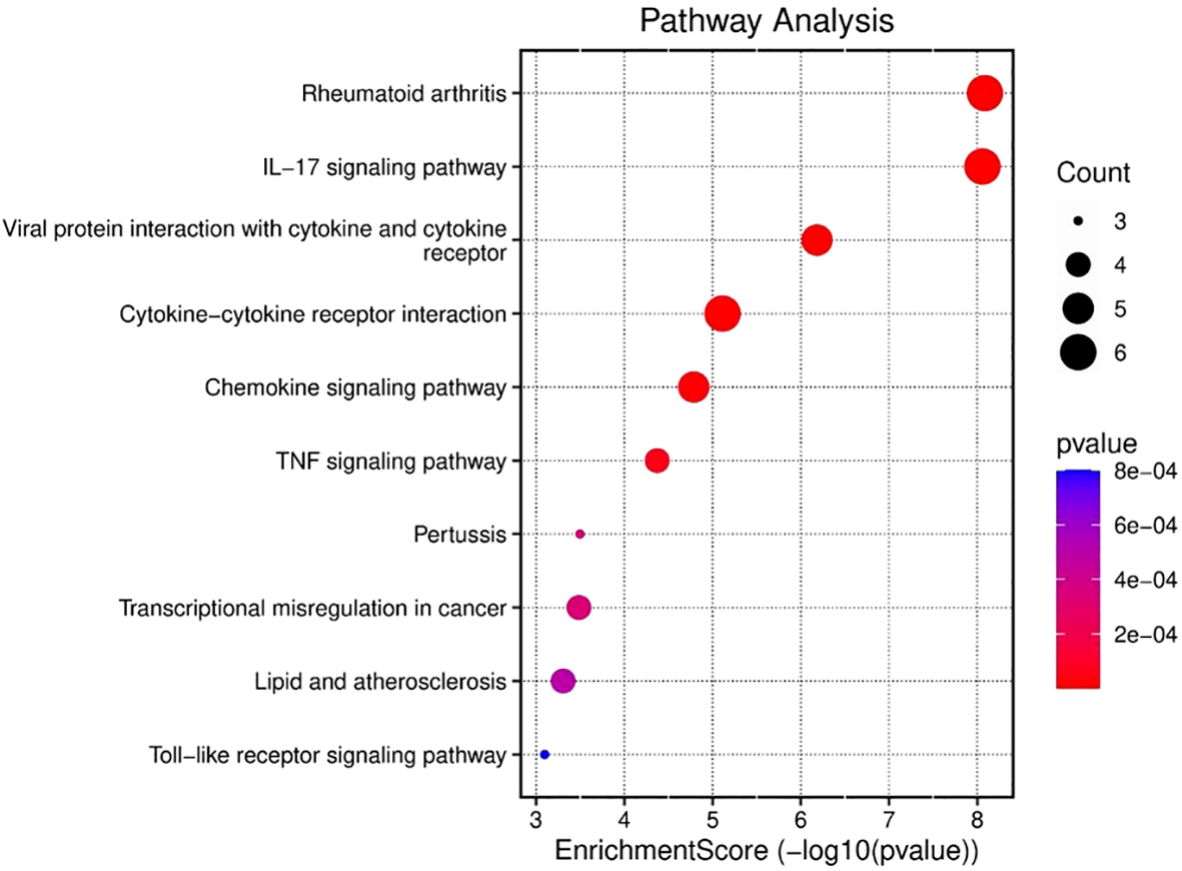
KEGG analysis was used to evaluate the ten most significant pathway enrichment terms for common genes across the three datasets.

Similarly, enrichment analysis of the KEGG pathway revealed correlations between the enriched pathways and interactions among gut microbiome and epigenetic modifications, such as the key pathways reported are IL-17 signaling pathway, the chemokine, cytokine–cytokine receptor interactions, cytokine receptors, the TNF, and pertussis associated pathways (**Figure 4**). The involvement of TNF and IL-17 signaling further emphasizes the importance of proinflammatory mediators, which are critical in driving immune cell recruitment, tissue inflammation and activation (Ruiz De Morales et al., 2020). Taken together, these results demonstrate that the gene set under study is strongly associated with immune regulatory networks, bridging innate and adaptive responses, and may provide insights into therapeutic targets for gut microbiome mediated epigenetic modifications in gastric cancer. Moreover, enriched pathways and functional terms support a model in which upregulated genes seem to be pivotal in structural maintenance, cell signaling, and immune response regulation, potentially contributing to the disease processes observed in GCs and the microbiota.

Several studies have revealed that TNF-α signaling is closely associated with inflammatory responses in tumor tissues. Proinflammatorycytokines such as CXCL12, TNF-α, and IL-6 are simultaneously induced within the tumor microenvironment, forming an interconnected cytokine network that amplifies inflammatory signaling. This network plays a crucial role in tumor progression, as some inflammatory mediators have been implicated in promoting tumorigenesis through mechanisms such as immune modulation, enhanced cell proliferation, and metastasis (Oshima et al., 2011, Oshima et al., 2014).

### 3.4 Module analysis and interpretation of the PPI network

**Figure 5:**
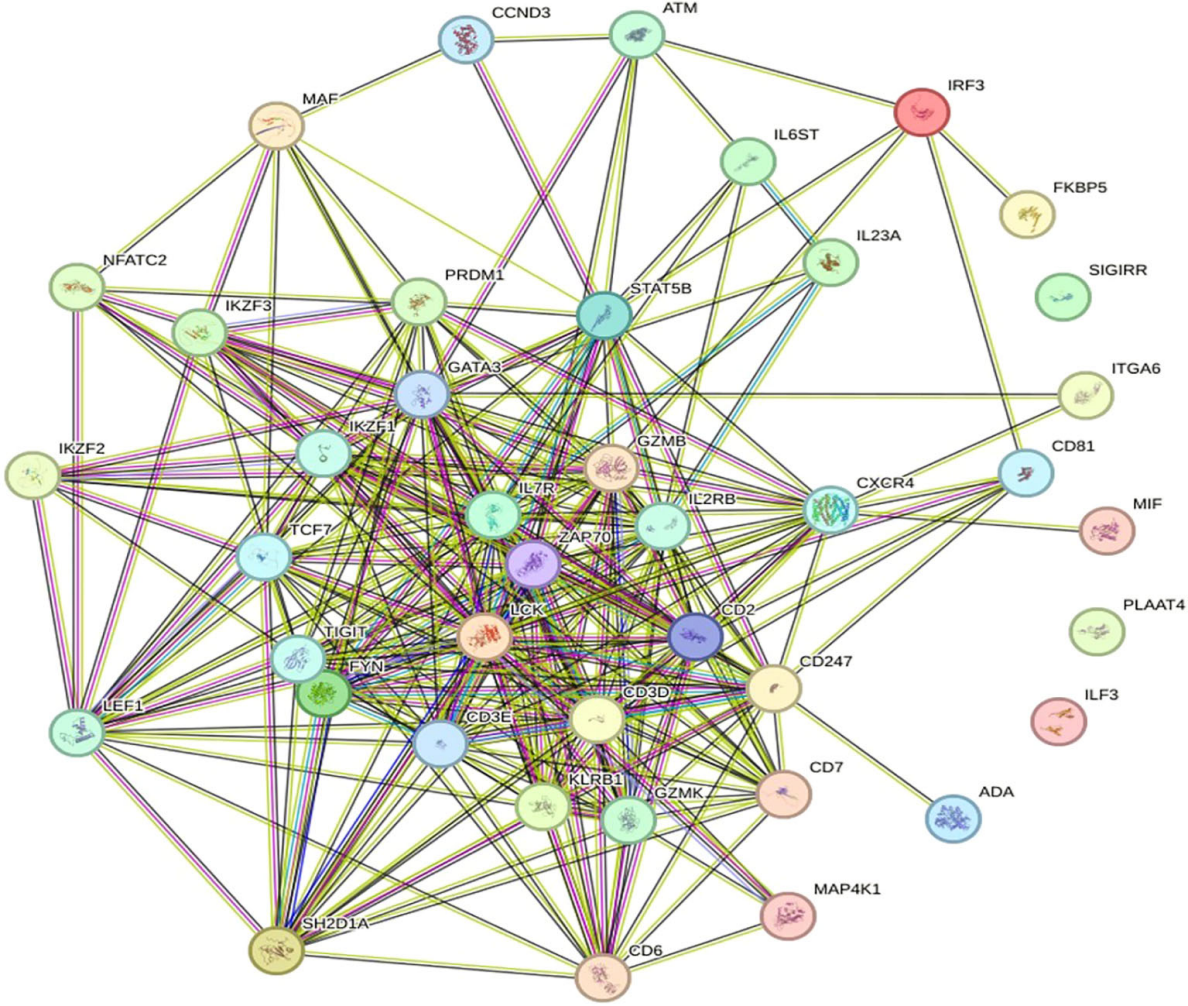
Diagram of the PPI network. Proteins are designated nodes in the PPI network analysis graph; a thicker connection denotes a higher score, and a thicker line denotes greater protein interaction.

The retrieved network has 35 nodes connected by 61 edges, resulting in an average node degree of approximately 3.49. One node is connected to an average of 3.49 additional nodes within the network. It is also thought to be 0. The limiting clustering coefficient is 503, which is the ratio of nodes that are close together in the network. The coefficient of betweenness centrality measures the moderate- to high-level clustering of the network, which means that nodes are more likely to belong to communities or clusters close to their surrounding nodes. Usually, the metrics provide meaningful data about the network’s connectivity and structure. This network shows a significant enrichment in PPIs that surpasses that expected by random chance. This finding potentially indicates a high probability that the observed interactions between proteins within the network are of significant biological or functional relevance. Moreover, the number of connections between nodes, similar to a random model with the same characteristics, is approximately 7, which confirms the organized nature of the network. A PPI enrichment p-value less than 1.0 e^-16^ further indicates the apparent nonrandom nature of protein interactions within the network and suggests complex biological or functional interrelationships between its components, as shown in **Figure 5**.

### 3.5 Identification of potential common hub genes associated with the three datasets

**Figure 6:**
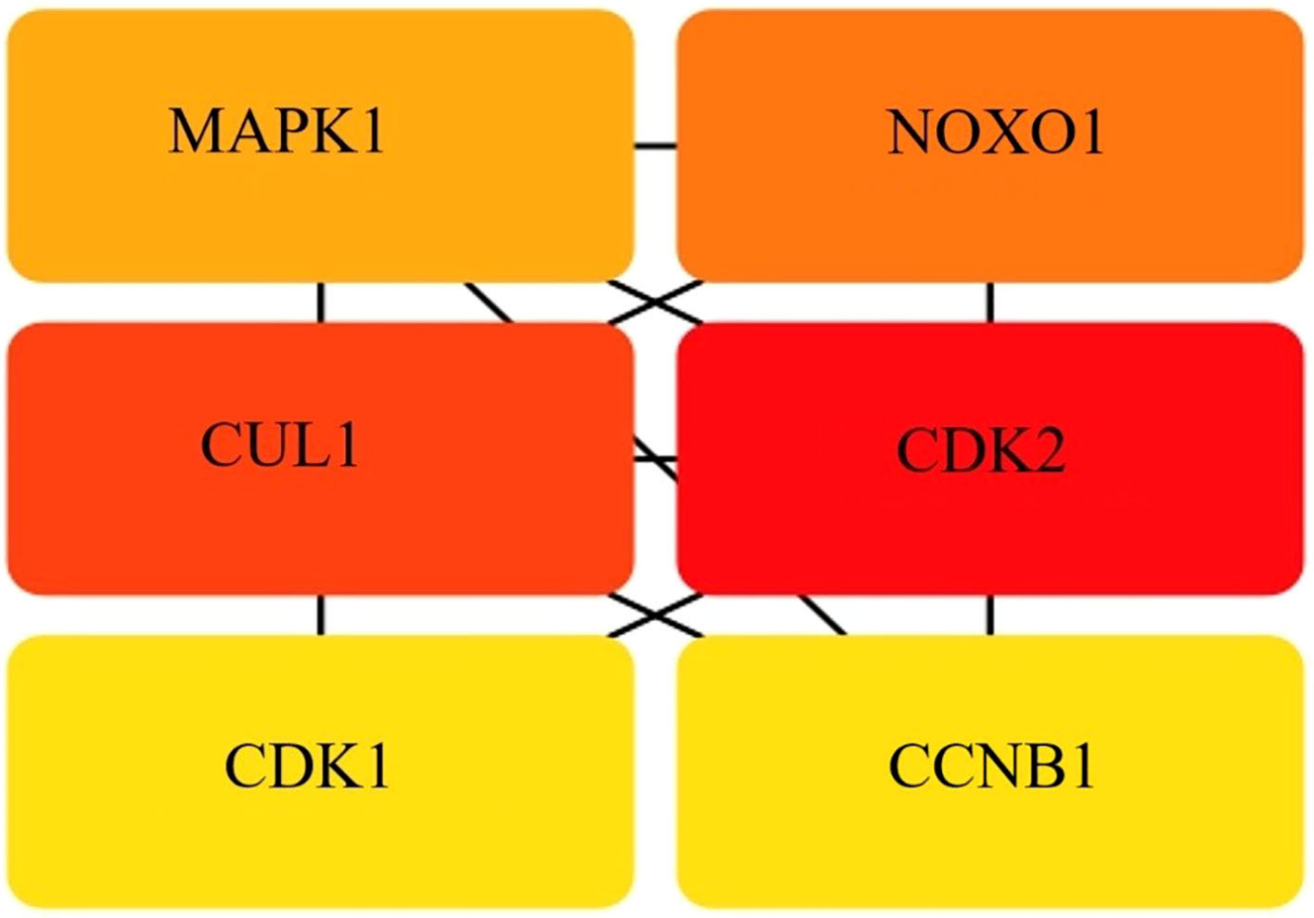
Hub gene identity. The intersection of 40 genes from 10 algorithms led to the identification of hub genes.

PPI networks were used to identify key nodes via topological analysis techniques such as DMNC, degree, MCC, EPC, MNC, bottleneck, eccentricity, radiality, closeness, stress, and betweenness, which were included in the CytoHubba plugin of Cytoscape. As a result, six hub genes were identified MAPK1, NOXO1, CUL1, CDK1, CDK2, and CCNB1. These genes were provided simultaneously with their aliases and primary functions, as shown in **Figure 6**.

Among the various functions and pathways, key elements, such as MAPK1, are strongly associated with GC and the microbiota. The interaction of TNFα with receptors on the cell surface initiates numerous signal transduction pathways, including those involving extracellular-signal-regulated kinases, three classes of mitogen-activated protein (MAP) kinases, p38 MAP kinases and cJun NH2-terminal kinases (JNKs). The signaling pathways associated with MAP kinase elicit a secondary response by increasing the expression of other inflammatory cytokines, including TNFα, which increases the biological activity of TNFα (Sabio and Davis, 2014; Bahar et al., 2023). Nicotinamide adenine dinucleotide phosphate (NADPH) oxidases (a NOX family enzyme) are often dysregulated in various human cancers, including gastric cancer (GC). Reactive oxygen species produced from NOX have been shown to play a role in gastric carcinogenesis and the progression of cancer (You et al., 2018). *H. pylori* infection induces chronic inflammation that contributes to GC, and researchers have reported that TNF-α signaling through TNFR1 is crucial for GCdevelopment and within the tumor microenvironment, it enables GC progression by inducing NOXO1 and Gna14, hence preserving tumor cells in an undifferentiated state (El-Omar et al., 2003; Mccoll et al., 2007).

Cullin1 (CUL1) another hub gene is the primary and most meticulously examined member of the cullin family. CUL1, a prominent scaffolding protein. It is also a crucial element of the SCF E3 ubiquitin ligase complex. Consequently, CUL1 governs the selective ubiquitination of certain proteins, facilitating several cellular activities, including cell cycle regulation and early embryonic development (Huang et al., 2018). CUL1 is upregulated in various cancers and linked to lymphatic and distant metastasis, and its degradation can induce epigenetic reprogramming, impacting tumorigenesis and cellular differentiation (Chen and Dent, 2014). CDKs and cyclins are key cell cycle regulators that regulate gastric cell growth, modifying the cell’s life cycle sequentially (Javed et al., 2023). Their dysregulation often leads to uncontrolled cell proliferation, a hallmark of cancer. Emerging evidence suggests that cyclins and CDKs interact with epigenetic modification systems, influencing gene expression, chromatin structure, and tumor progression (Suski et al., 2021), and the identification of CDK1 and CDK2 the distinct example of epigenetic modification associated with GC. Another important gene identified as a hub gene in this study was cyclin B1 (CCNB1). CCNB1 is a key regulator of the G2/M transition in the cell cycle and primarily interacts with CDK1 to drive mitosis. Aberrant expression of CCNB1 is frequently observed in various cancers, and accumulating evidence suggests that epigenetic modifications play crucial roles in its dysregulation (Wang et al., 2014; Javed et al., 2023). Moreover, it has also been reported as a potential biomarker for various cancer types, such as pancreatic ductal adenocarcinoma (PDAC) (Zeng and Fan, 2022).

These findings emphasize the potential shared molecular pathways between GC, GC linked with Hp and epigenetic modifications and provide a deeper understanding of the interplay between infectious diseases and cancer. Overall, the literature validates that RNA - seq data and hub gene functionalities potentially offer promising avenues for both GC and microbiota prognostic applications and the development of novel anticancer therapies.

### 3.6 TF and TF–miRNA regulatory network analysis of the hub genes

**Figure 7:**
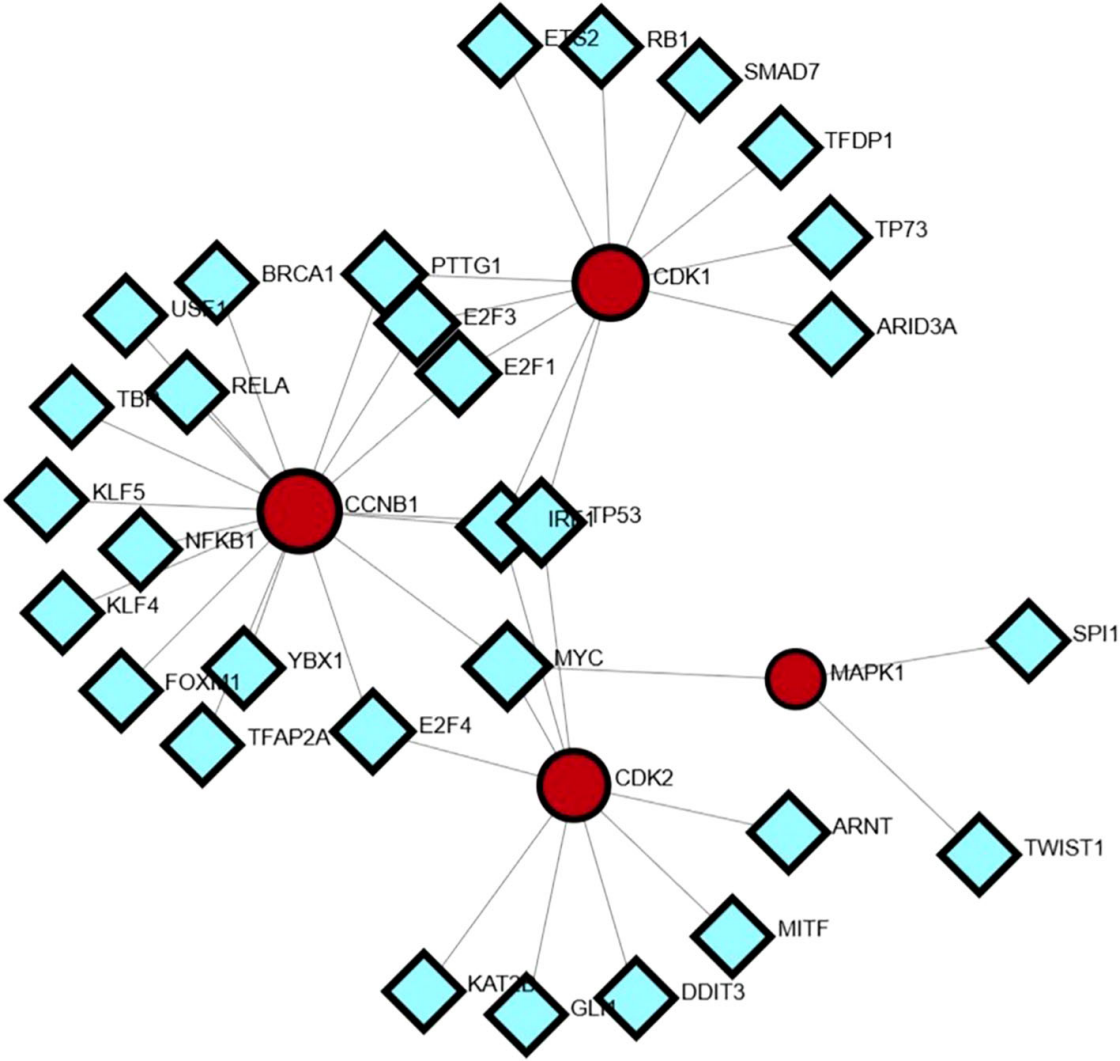
Transcription factor–hub gene regulatory network.

A transcription factor (TF) regulatory network was constructed for the hub genes via the NetworkAnalyst platform. A transcription factor (TF) regulatory network was constructed for the hub genes via the NetworkAnalyst platform. These findings indicate that key hub genes, CCNB1, CDK1, CDK2, and MAPK1, are under the control of multiple TFs. For example, CCNB1 exhibited the greatest number of TF interactions with BRCA1, FOXM1, RELA, NFKB1, and E2F family members, emphasizing its importance in proliferation and cell cycle regulation. CDK1 is regulated by TFs such as RB1, SMAD7, TP73, and E2F1/E2F2, and CDK2 which are known to have regulatory associations with MYC, GLI1, MITF, and KAT2B. The findings are consistent with their role in mediating genetic networks implicated in the progression of various cancers, particularly gastric cancer (Pellarin et al., 2025). Although MAPK1 was targeted by fewer TFs (e.g., SPI1, TWIST1, and ARNT), its role as a signaling hub suggests that transcriptional modulation could exert broad downstream effects across multiple cellular pathways (Kubatka et al., 2025) (**Figure 7**).

**Figure 8:**
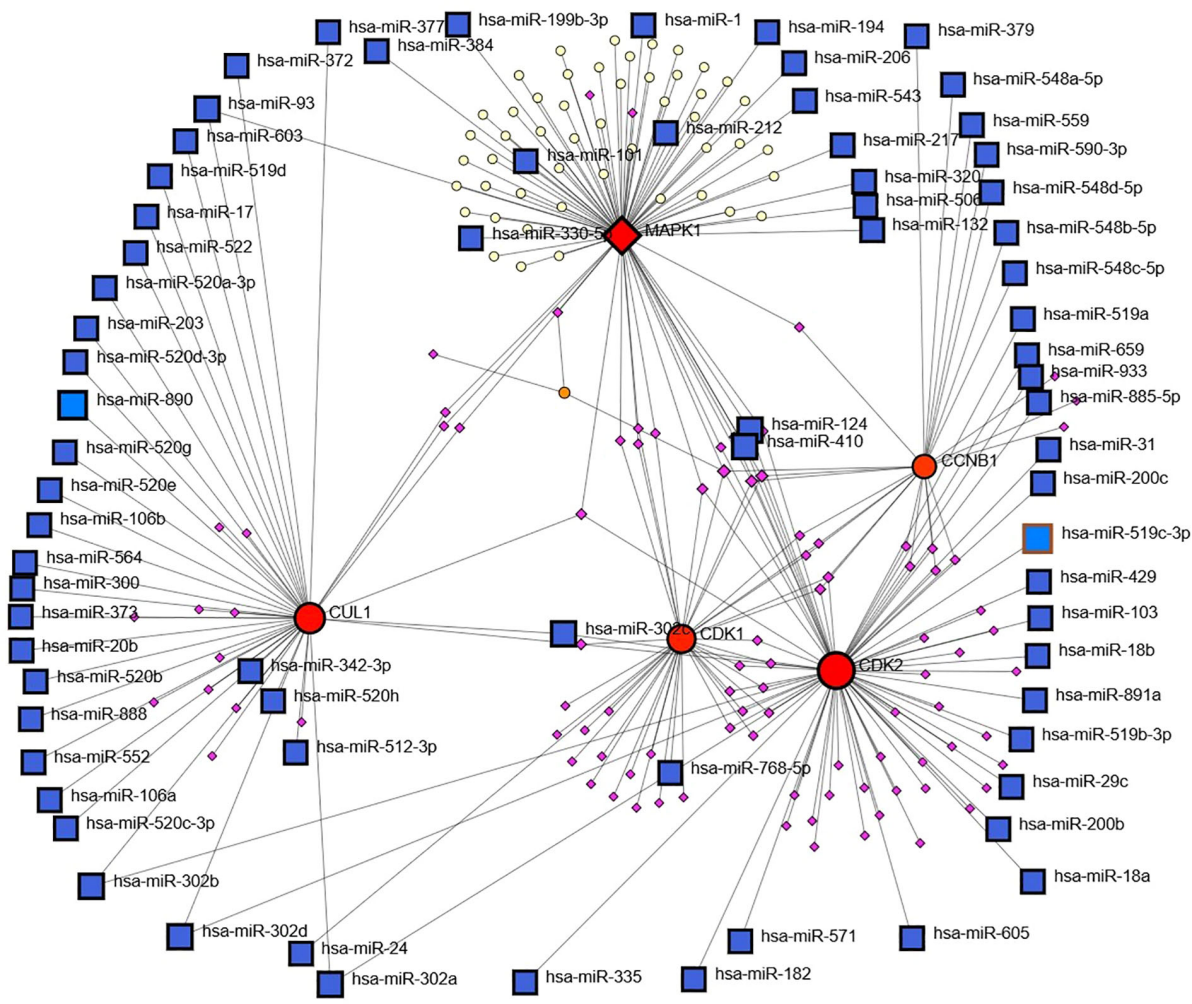
Transcriptionfactor (TF) and miRNA regulatory network analysis via NetworkAnalyst.

The miRNA–mRNA network revealed extensive posttranscriptional regulatory interactions, with hub genes such as CDK2, CCNB1, CUL1, CDK1, and MAPK1 exhibiting high connectivity to numerous miRNAs. Among these, CDK2 and CUL1 were targeted by the largest number of miRNAs, suggesting their roles as major regulatory nodes. Several miRNAs, including hsa-miR-16, hsa-miR-330, hsa-miR-17, hsa-miR-200 family members, and hsa-miR-520 family members, displayed multi targeting properties, indicating their potential function as master regulators in the studied biological context. The dense connectivity of these miRNAs with multiple hub genes suggests a coordinated post-transcriptional control mechanism influencing key cellular pathways **Figure 8**. Integration of both networks revealed that the hub genes are subject to dual-layer regulation at the transcriptional level via TFs and at the posttranscriptional level via miRNAs. This combined regulatory framework points to a tightly controlled gene expression system, where worries at either regulatory layer could significantly impact disease-associated pathways. This integrated network analysis provides a comprehensive view of the molecular control architecture, identifying candidate miRNAs and TFs that could serve as potential therapeutic or diagnostic targets alongside the hub genes themselves.

### 3.7 Validation of the hub genes and common genes

**Figure 9:**
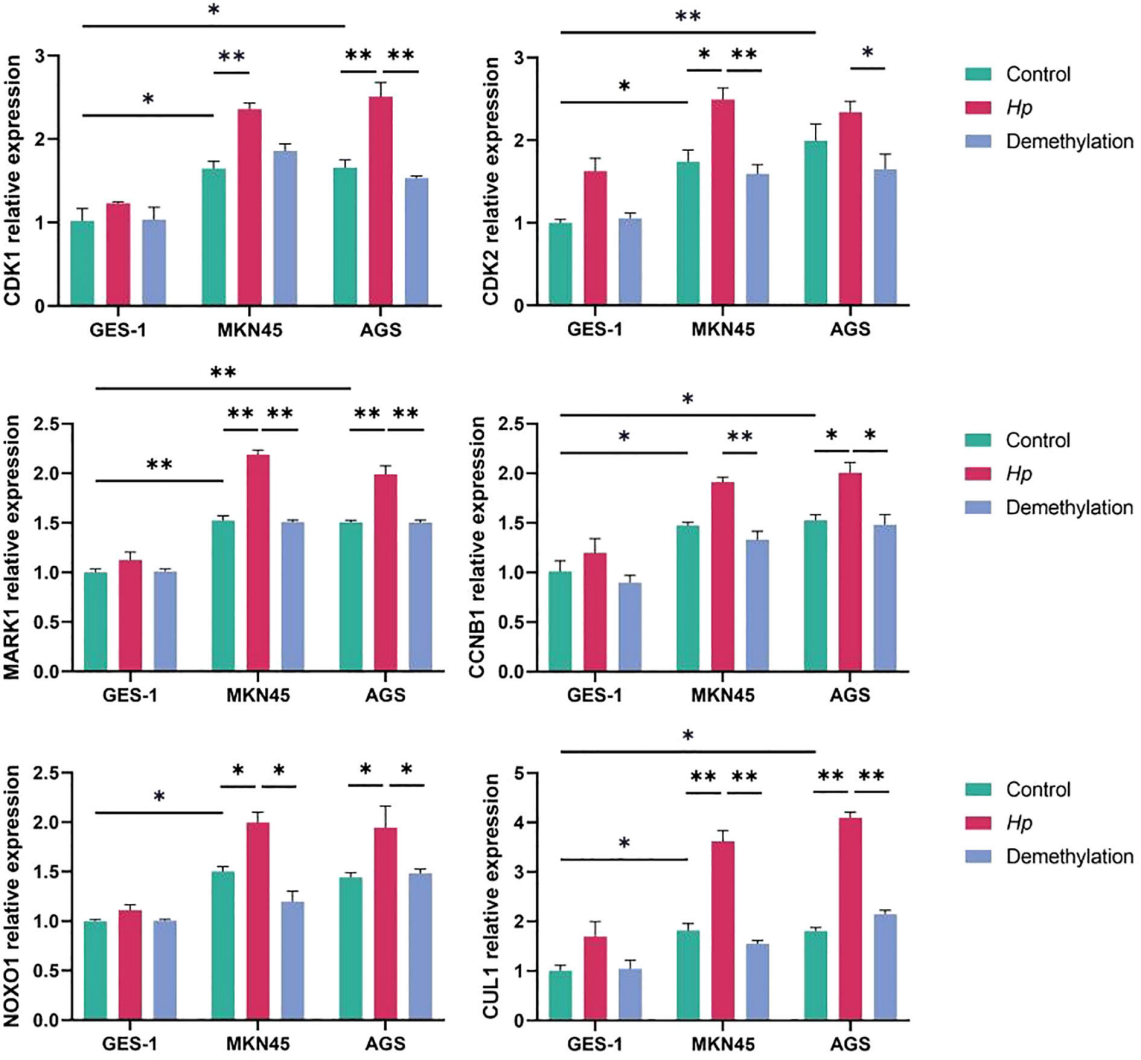
Validation of gene expression via qRT–PCR via the ΔΔCt method. *p < 0.05, **p < 0.01.

Changes in the transcription of the of hub genes MAPK1, NOXO1, CUL1, CDK1, and CCNB1 were detected in cell lines, i.e., AGS, which are well-differentiated human gastric adenocarcinoma cells, and the normal gastric epithelial cell line GES-1 via quantitative RT–PCR. The results demonstrated that the relative gene expression levels of MKN45 and AGS cell lines were greater than those in the GES-1 cell line in the control. Compared with the control group, *H. pylori* infection significantly increased the mRNA expression levels of core genes in AGS and MKN45 cells (**Figure 9**). In contrast, minimal changes were observed in GES-1 cells. Pretreatment with 5-Aza-dC markedly attenuated *H. pylori*-induced upregulation in cancer cells, reducing the expression levels of genes. The experimental validation conducted in this study provides functional support for our bioinformatic predictions, demonstrating that *H. pylori* infection significantly upregulates the expression of identified hub genes in gastric cancer cells. The markedly attenuated effect observed following 5-Aza-dC pretreatment indicates that DNA methylation plays a crucial role in mediating *H. pylori*-induced transcriptional activation which is in line with previous studies (Valenzuela et al., 2015). These findings not only confirm the reliability of our multiomics analysis but also highlight the importance of epigenetic mechanisms, particularly DNA methylation, in *H. pylori*-associated gastric carcinogenesis. The differential response between gastric cancer cells and normal gastric epithelial cells suggests that these genes may represent promising targets for the development of epigenetic-based therapeutic strategies against *H. pylori*-related gastric cancer.

## 4 Conclusions

An integrated bioinformatics study revealed six hub genes—MAPK1, NOXO1, CUL1, CDK1, CDK1 and CCNB1 and the same genes were common to GC and gut microbiome. The identification of hub genes in our study elucidated the roles of inflammation, cell adhesion, and cell proliferation. These findings emphasize the shared molecular pathways between GC, *H. pylori*-associated GC, and epigenetic modifications, offering insights into the infection–cancer link. While, the integrated network analysis identifies hub genes, miRNAs, and TFs as potential diagnostic and therapeutic targets, supporting the use of RNA-seq data for prognostic applications and novel anticancer strategies. Moreover, findings of this study necessitate comprehensive analysis utilizing animal models and clinical samples to elucidate the molecular mechanisms of GC pathogenesis; clarify the roles of CDK1, CDK1, MAPK1, NOXO1, CUL1 and CCNB1 in GC; and identify innovative and promising targets for the early diagnosis and treatment of GC.

## Data Availability

The datasets presented in this study can be found in online repositories. The names of the repository/repositories and accession number(s) can be found in the article/**Supplementary Material**.
