# Supplementary material for: Gut microbiome-mediated epigenetic modifications in gastric cancer: a comprehensive multiomics analysis
Source: Front Cell Infect Microbiol. 2025 Oct 16;15:1585881. doi: 10.3389/fcimb.2025.1585881 (PMC12571729; doi:10.3389/fcimb.2025.1585881)
Supplement: Supplementary file 1 [file Table1.docx]

Supplementary Material

# Table S1

| **Table S1. Compsotion of the gut microbiome in GC pateints reported till 2024** | | | | |  |
| --- | --- | --- | --- | --- | --- |
| **Sample Size** | **Origin of Sample** | **Sequencing Methods** | **Decreased bacteria Composition in GC** | **Increased Non-Helicobacter bacteria Composition in GC** | |
|  |  |  |  |  | |
|  |  |  |  |  | |
| 12 GC patients and 20 functional | Gastric antral biopsies | 16S rRNA gene amplicon sequencing | *Methylobacterium* | *Lactococcus, Veilonella*, | |
| dyspepsia |  |  |  | *Fusobacterium* | |
| 160 GC patients | Gastric tumor tissues and matched non-malignant tissues | 16S rRNA gene amplicon sequencing | – | *Helicobacter, Proteobacteria* | |
|  |  |  |  | *Helicobacter, Proteobacteria* and *Firmicutes* | |
| 81 chronic gastritis, 54 GC patients | Gastric biopsies and | 16S rRNA gene profiling | *Helicobacter* and *Neisseria* | *Phyllobacterium* and *Achromo-* | |
|  | non-malignant tissues adjacent to the tumor |  |  | *Bacter*, *Xanthomonadaceae*, *Enterobacte-Riaceae, Lactobacillus, Clostridium, Rhodococcus* | |
| 21 SG, 23 AG, 17 IM, 20 GC patients | Gastric biopsies and matched | 16S rRNA gene amplicon sequencing | *Vogesella*, *Candidatus_Portiera*, *Comamonadaceae*, *Acinetobacter* | *Peptostreptococcus* | |
|  | non-malignant tissues |  |  | *stomatis, Streptococcus anginosus, Parvimonas micra*, | |
|  |  |  |  | *Slackia exigua, Dialister pneumosintes* | |
| 6 GC and 5 SG patients | Gastric wash samples | shotgun metagenomic sequencing | *Sphingomonadaceae* | *Neisseria*, | |
|  |  |  |  | *Alloprevotella*, *Aggregatibacter* | |
| 276 patients with GC | Gastric tumor tissues and matched normal tissues and peritumoral tissues | 16s rRNA gene sequencing | – | *Prevotella melaninogenica*, *Streptococcus anginosus*, *Propionibacterium acnes* | |
| 62 GC patients | Gastric tumor tissues and matched non-malignant tissues | 16s rRNA gene sequencing | – | Genus *Streptococcus*, *Peptostreptococcus*, | |
|  |  |  |  | *Prevotella*, *Prevotella_7 Acinetobacter*, *Bacillus*, *Selenomonas*, | |
|  |  |  |  | *Lachnoanaerobaculum* | |
| 64 GC patients | Gastric tumor tissues and matched normal tissues and peritumoral tissues | bacterial genomic DNA sequencing | *Staphylococcus* and *Corynebacterium* | *Streptococcus*, | |
|  |  |  |  | *Peptostreptococcus*, *Lactobacillus*, *Bifidobacterium*, *Neisseria*, | |
|  |  |  |  | *Veillonella*, *Shewanella* | |
| 57 *Hp* positive patients, 58 *Hp* negative patients after treatment, and 49 *Hp* negative patients | Gastric biopsies | 16s rRNA gene sequencing | *Proteobacteria*, *Epsilonproteobacteria*, *Campylobacterales*, *Helicobactera*ceae, *Helicobacter* | *Cyanobacteria/Chloroplast, Bacteroidetes, Fusobacteria, Actinobacteria*, *Firmicutes* | |
|  | Stool samples |  | *Bacteroidales* | *Clostridiales* and *Bifidobacterium* | |
| 120 noncancer | Gastric biopsies and matched non-malignant tissues | 16S rRNA gene amplicon sequencing | – | *Enterococcus*, *Lactobacillus*, *Firmicutes* | |
| patients, 48 GC patients |  |  |  |  |  |
| 22 patients with dyspepsia, 12 GC patients | Gastric antral biopsies | 16S rRNA gene-targeted amplicon sequencing | *Actinomyces* spp. | *Streptococcus* | |
| 30 healthy controls (HC), 21 non- | Gastric biopsies and gastric tumor tissues | 16s rRNA gene sequencing | Aerobic and facultatively anaerobic bacteria | *Lactobacillus*, *Streptococcus*, *Prevotella*, *Veillonella* | |
| atrophic chronic gastritis (CG), 27 IM, 25 IN, and 29 |  |  |  |  |  |
| GC patients |  |  |  |  |  |
| 375 tumors and 27 matched normal tissues | Gastric cancer samples and around tissues | TCGA and GTEx data analysis | *Helicobacter* | *Bacillus, Parasutterella, Brevibacillus, Fusobacterium, Enterobacter, Cloacibacterium*, *Suterella* | |
|  |  |  |  | *Firmicutes* | |
| 50 patients with a history of gastrectomy for gastric cancer and 56 control patients | Stool samples | shotgun metagenomics sequencing | – | *Streptococcus* spp.*, Prevotella* spp.*, Veillonella* spp., *Lactobacillus* spp. | |
| 61 healthy individuals, 83 patients with GC | Stool samples | 16s rRNA gene sequencing | – | *Lactobacillus*, *Megasphaera* | |
| 35 healthy people, 38 patients with GC | Stool samples | Fecal samples DNA sequencing | *Faecaliberium*, *Roseburia*, *Lachnospira*, *Anaerostipes* | Genera *Enterobacteriaceae*, *Streptococcaceae*, *Desulfovibrio* | |
| 32 superficial gastritis (SG) patients, 18 GC patients | Gastric biopsies; gastric tumor tissues and matched non-malignant tissues | 16s rRNA gene sequencing | *Fusobacterium* spp. | *Dialister* spp., *Helicobacter* spp., *Lactobacillus* spp.*, Rhodococcus* spp.*, Rudaea* spp., *Sediminibacterium* spp. | |
| 43 | Gastric biopsies | 16s rRNA gene sequencing | *Lactobacillus* and *Bifidobacteria* | *Phyllobacteriaceae*, *Enhydrobacter*, *Moryella* | |
| participants |  |  |  |  |  |
| 37 patients with GC | Gastric tumor tissues and matched normal tissues | 16s rRNA gene sequencing | *Helicobacter* | *Lactobacillus, Streptococcus, Acinetobacter, Prevotella, Sphingomonas, Bacteroides*, *Fusobacterium* | |
| 13 proximal and16 distal GC patients | Gastric tumor tissues and matched normal tissues | 16S rRNA amplicon sequencing | In proximal GC: *Rikenellaceae_RC_gut_group* | In proximal GC: *Helicobacter* | |
|  |  |  | In distal GC: *Methylobacterium-Methylorubrum* | In distal GC: *Helicobacter* | |
| 5 patients each of non-atrophic gastritis (NAG), IM and intestinal-type GC | Gastric biopsies | 16S rRNA microarray | TM7, *Porphyromonas* and *Neisseria* | *Lactobacillus coleohominis* and *Lachnospiraceae* | |
| 5 dyspeptic control patients and 10 GC patients | Gastric biopsies | T-RLFP, 16S rRNA gene sequencing | – | *Streptococcus* | |
